# Letter from the Editor in Chief

**DOI:** 10.19102/icrm.2018.090609

**Published:** 2018-06-15

**Authors:** Moussa Mansour


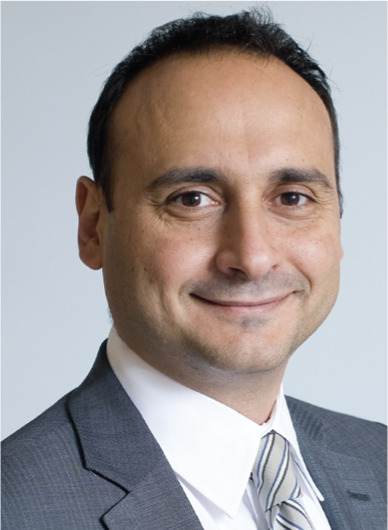


Dear Readers,

Year after year, the annual scientific meeting of the Heart Rhythm Society continues to offer cardiac electrophysiologists from across the globe access to the latest and most interesting discoveries in research and education. This year’s meeting, which was held in Boston in early May, was no exception.

Perhaps the most exciting presentation during the meeting this year was on the results of the Catheter Ablation versus Antiarrhythmic Drug Therapy for Atrial Fibrillation (AF) (CABANA) trial. This multinational randomized study enrolled more than 2,000 patients, who were randomized to undergo either catheter ablation or drug therapy and/or rate control between 2009 and 2016. The primary endpoint was a composite of death, disabling stroke, serious bleeding, or cardiac arrest. The key finding was that ablation reduced mortality or cardiovascular hospitalization by 17% versus drug therapy. Also, there were reductions of 33% and 40% in the primary endpoint and mortality, respectively, when patients underwent ablation. However, there were no significant differences in these parameters between groups when intent-to-treat analysis was performed, probably resulting from a significant rate of crossover. Additional key findings in the study included an increased likelihood of remaining in normal sinus rhythm and a better quality of life for patients who underwent ablation; I believe the most important result is that ablation is a safe procedure and associated with only a very low rate of complications.

CABANA is a landmark study that is expected to leave a permanent mark on the field of AF ablation. It confirmed our belief that catheter ablation is a superior treatment to the use of pharmacological agents and corroborated the findings of many other randomized clinical trials, most recently the Ablation versus Amiodarone for Treatment of AF in Patients with Chronic Heart Failure and an Implantable Cardioverter-defibrillator (AATAC-AF)^1^ and Catheter Ablation versus Standard Conventional Treatment in Patients with Left Ventricular Dysfunction and AF (CASTLE AF)^2^ studies. Other randomized investigations that have suggested the superiority of catheter ablation include the Ablation for Paroxysmal AF (APAF2) trial,^3^ the NAVISTAR^®^ THERMOCOOL^®^ Catheter for the Radiofrequency Ablation of Symptomatic Paroxysmal AF study,^4^ the Sustained Treatment of Paroxysmal AF (STOP AF) trial,^5^ and Catheter Ablation versus Antiarrhythmic Drugs for AF (the A4 study).^6^

Sincerely,


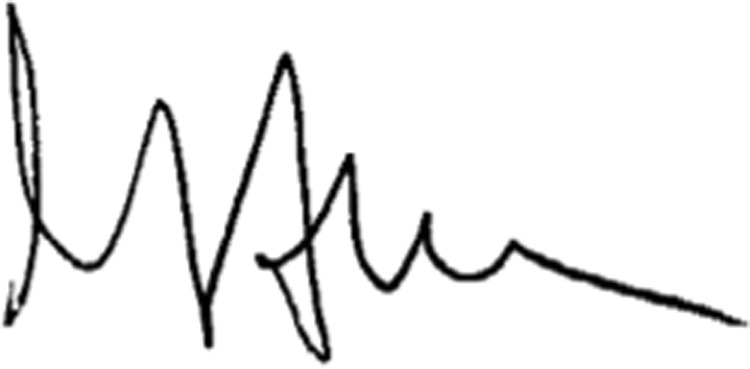


Moussa Mansour, MD, FHRS, FACC

Editor in Chief

The Journal of Innovations in Cardiac Rhythm Management

MMansour@InnovationsInCRM.com

Director, Atrial Fibrillation Program

Jeremy Ruskin and Dan Starks Endowed Chair in Cardiology

Massachusetts General Hospital

Boston, MA 02114
